# On-Site Sensor Recalibration of a Spinning Multi-Beam LiDAR System Using Automatically-Detected Planar Targets

**DOI:** 10.3390/s121013736

**Published:** 2012-10-12

**Authors:** Chia-Yen Chen, Hsiang-Jen Chien

**Affiliations:** Department of Computer Science and Information Engineering, National University of Kaohsiung, 700, Kaohsiung University Rd. Nan Tzu Dist., Kaohsiung, Taiwan

**Keywords:** on-site calibration, LiDAR system, 3-D reconstruction, plane detection

## Abstract

This paper presents a fully-automated method to establish a calibration dataset from on-site scans and recalibrate the intrinsic parameters of a spinning multi-beam 3-D scanner. The proposed method has been tested on a Velodyne HDL-64E S2 LiDAR system, which contains 64 rotating laser rangefinders. By time series analysis, we found that the collected range data have random measurement errors of around ±25 mm. In addition, the layered misalignment of scans among the rangefinders, which is identified as a systematic error, also increases the difficulty to accurately locate planar surfaces. We propose a temporal-spatial range data fusion algorithm, along with a robust RANSAC-based plane detection algorithm to address these issues. Furthermore, we formulate an alternative geometric interpretation of sensory data using linear parameters, which is advantageous for the calibration procedure. The linear representation allows the proposed method to be generalized to any LiDAR system that follows the rotating beam model. We also confirmed in this paper, that given effective calibration datasets, the pre-calibrated factory parameters can be further tuned to achieve significantly improved performance. After the optimization, the systematic error is noticeable lowered, and evaluation shows that the recalibrated parameters outperform the factory parameters with the RMS planar errors reduced by up to 49%.

## 1. Introduction

The need to acquire 3-D information of physical environments has escalated in the last decade. With the rapid advances of Light Detection and Ranging (LiDAR) technology, laser-based active scanning has become a fast, accurate, and popular measurement tool. Stationary terrestrial LiDAR systems in the market such as the Reigl VZ-1000 and Optech ILRIS-3D units, are capable of delivering industrial-level accuracy with errors lower than 10 mm. For the real-time acquisition of panoramic range information, multiple laser rangefinders are attached to a motor, forming a multi-beam LiDAR system. The Velodyne HDL-64E S2, which is equipped with 64 laser emitter-sensor pairs to deliver dynamic panoramic point cloud at 10 Hz within the working range from 0.9 to 120 m, is such a high-definition LiDAR system. Although the LiDAR system was originally designed for the DARPA Grand Challenge and is often used in applications such as mobile navigation and autonomous vehicles ([1,2]), which require intermediate accuracy, recent research (e.g., [3–6]) points out that the model has the potential to be a promising solution for static 3-D mapping, which requires higher accuracy.

**Figure 1.**
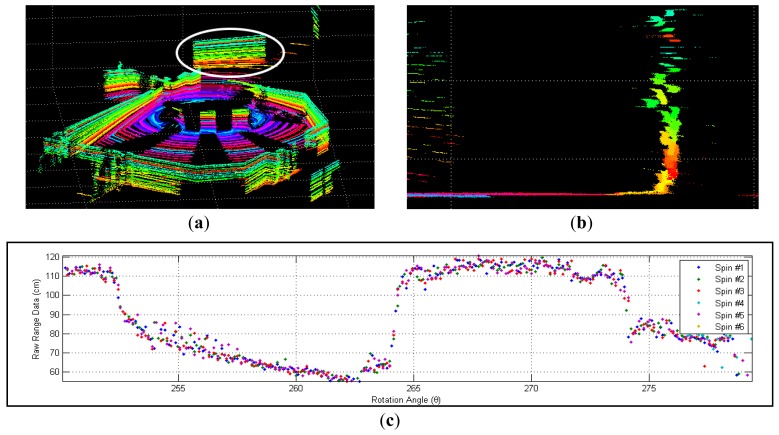
(**a**) point cloud of corridor; colour-coded to differentiate laser sources; (**b**) A close look at the marked wall; (**c**) range data of laser #40 returned in six subsequent spins.

In our experiment, we have found that the tested Velodyne HDL-64E S2 achieves an average RMS error of about 2.5 cm. Close examination of the recorded range data has revealed two major problems. The first issue is the layered misalignment of scans on planar surfaces, as shown in Figure 1(b). This phenomenon is due to the use of inaccurate intrinsic parameters that include the orientation and offset of each laser rangefinder for the conversion of raw sensor readings to 3-D point cloud. In addition, we have found that the data returned from each range sensor at a fixed rotation angle fluctuates over time within an interval, as shown in Figure 1(c). This may be caused by the quantization error of rotation angle, random measurement error intrinsic to the time-of-flight data, and motor vibrations.

The intrinsic parameters are calibrated by the manufacturer using a plane placed at 25.04 m. The 3-D coordinates of a point becomes less accurate as it moves away from this distance. To address the aforementioned cause of systematic error, we have to further optimize the parameters for a certain range using data acquired in the scanned field. This is known as online or on-site calibration [7]. In this work we propose an automatic strategy to perform on-site recalibration of the intrinsic parameters.

**Figure 2.**
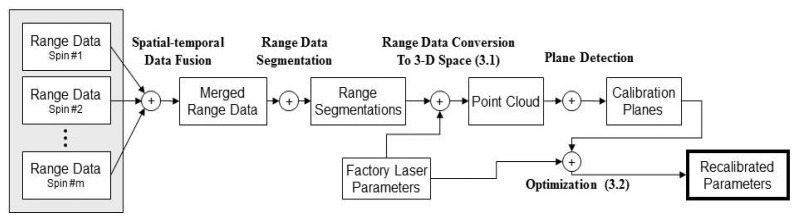
Process of the proposed automatic on-site recalibration method.

Figure 2 depicts the tasks performed by the proposed method. First, the system records a short range data stream of the surroundings. The collected range data are then merged to produce a more reliable dataset. Afterward, the range data are segmented and the points that are less likely to be on a plane are filtered out. In next stage, we convert the processed range data to 3-D points and apply a robust plane detection algorithm on the point cloud to establish the calibration dataset, which is used to improve the intrinsic parameters of the LiDAR system. These tasks are detailed in the following sections.

There are two major contributions in this paper. First, we formulate an alternative geometric interpretation of sensory data using linear parameters, instead of the nonlinear form specified by the manufacturer. The formulated linearly parameterized form has less correlation and achieves lower RMS error after calibration, as will be shown by the experiments. Second, and the more important contribution, is that we have implemented a framework for automatic on-site calibration using planes that exist within the scene. The calibration process can be easily adapted to other types of LiDAR and has been proven to achieves an RMS error lower than the factory provided calibration parameters.

The rest of this paper is organized as follows: in Section 2, related research is surveyed. InSection 3, the mathematical models of the conversion of range data to 3-D space and the adjustment of parameters are given. In Section 4, we propose a data fusion algorithm and the automatic establishment of calibration dataset. Experimental results are discussed in Section 5, and Section 6 concludes this paper.

## 2. Related Work

There has not been a lot of work published in the literature specific to the calibration of the tested Velodyne LiDAR system since the device is relatively new. In [3], the sensor is placed in various positions with its X-Z or Y-Z planes parallel to a wall. The range data returned by 16 selected rangefinders are then used to optimize a part of the laser parameters (the details of these parameters will be described in the next section). Although the restricted positional condition simplifies the objective function to the variances of 1-D coordinates, the resulting model is not applicable to adjust the remaining parameters. These parameters are therefore ignored in their work.

The systematic error caused by the inaccurate intrinsic parameters is also examined in [4]. Unlike other work, their adjustment does not use factory parameters at all. The scanner is placed in the centre of a precisely-made calibration object, and the parameters are initially estimated from a scan of the object. The objective function is then minimized using the Levenberg-Marquardt algorithm. In the optimized case the measurement error is 1.56 cm.

In [5], the factory intrinsic parameters are taken as an initial guess and iteratively refined using the Gauss-Helmert algorithm. The calibration data are collected from multiple sites, and the Euclidean transformations between different scanner locations are taken into account during optimization. However, the parameters are optimized against a definition of error that is biased to the distance of calibration target. The improvement of 25% in flatness error over factory parameters is reported with the final RMS error of 1.3 cm. In a follow-up paper [6], the temporal stability of the LiDAR system is analyzed. The authors have also extended the manufacturer-defined geometric model to include the error of the rotation angle measurements.

By referencing previous work, the proposed method also utilizes planar targets for calibration. However, we adopt a linear representation of intrinsic parameters. Moreover, instead of conducting laboratory calibrations, we deploy an automatic mechanism to establish and process calibration data on-site. The proposed method allows the LiDAR system to autonomously adjust its intrinsic parameters while operating online.

## 3. Optimization Model

Based on the geometric interpretation of the range data, a non-linear optimization model can be derived given some observed scene planes. This section first introduces an alternative model for the conversion of range data; then describes the objective function which minimizes plane deviations in terms of quadratic error.

### 3.1. Conversion of Raw Data to 3-D Cartesian Coordinates

**Figure 3.**
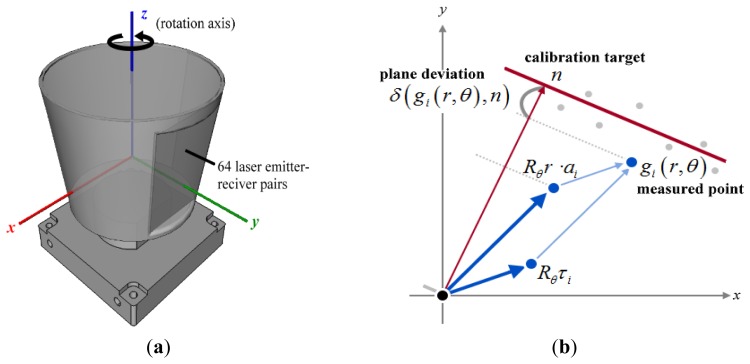
(**a**) coordinate system of the Velodyne LiDAR system; (**b**) geometric relation of laser intrinsic parameters in linear representation, a calibration plane, and the residual.

The Velodyne HDL-64E S2 contains 64 laser emitter-sensor pairs which are rigidly attached to a rotating motor, as depicted in Figure 3(a). In this work we define the LiDAR coordinate system to rotate about the z-axis and have the y-axis as the initial direction the scanner points. A raw reading is denoted by (*θ*, *r*), where *θ* is the rotation angle at which time-of-flight data *r* is measured. According to the documentation [8], for the *i*-th laser rangefinder, a reading (*r*, *θ*) is converted to 3-D coordinates by:

(1) $$g_{i}(r,\theta )=\left[\begin{array}{c} x\\ y\\ z \end{array}\right]=\left[\begin{array}{c} \left(m_{i}r+\Delta r_{i})\cos\alpha_{i}(\sin\theta \cos\theta_{i}+\cos\theta \sin\theta_{i})-\Delta x_{i}(\cos\theta \cos\theta_{i}-\sin\theta \sin\theta_{i}\right)\\ \left(m_{i}r+\Delta r_{i})\cos\alpha_{i}(\cos\theta \cos\theta_{i}+\sin\theta \sin\theta_{i})+\Delta x_{i}(\sin\theta \cos\theta_{i}+\cos\theta \sin\theta_{i}\right)\\ (m_{i}r+\Delta r_{i})\sin\alpha_{i}+\Delta z_{i} \end{array}\right]$$

where sensor-specific parameters include Δ*x_i_*, the horizontal offset; Δ*z_i_*, the vertical offset; Δ*r_i_*, the range offset; *θ_i_*, the azimuth angle; α*_i_*, the elevation angle and *m_i_*, the scaling factor are involved. These parameters are intrinsic to the sensor and remain fixed throughout the measurement. Their default values are calibrated by the manufacturer.

Although it is obvious that the described conversion is not linear, the parameters can actually be interpreted in a linear form by the following steps. First, extract the rotation matrix from the right-hand side of Equation (1) which gives:

(2) $$\left[\begin{array}{ccc} \cos\theta & -\sin\theta & 0\\ \sin\theta & \cos\theta & 0\\ 0 & 0 & 1 \end{array}\right]\;\left[\begin{array}{c} (m_{i}r+\Delta r_{i})\cos\alpha_{i}\sin\theta_{i}-\Delta x_{i}\cos\theta_{i}\\ (m_{i}r+\Delta r_{i})\cos\alpha_{i}\cos\theta_{i}+\Delta x_{i}\sin\theta_{i}\\ (m_{i}r+\Delta r_{i})\sin\alpha_{i}+\Delta z_{i} \end{array}\right]$$

Let *R_θ_* represent the rotation matrix of rotating by *θ* degrees about *z*-axis of the LiDAR coordinate system, Equation (2) can be rewritten as:

(3) $$R_{\theta }\left[r\cdot m_{i}\left(\begin{array}{c} \cos\alpha_{i}\sin\theta_{i}\\ \cos\alpha_{i}\cos\theta_{i}\\ \sin\alpha_{i} \end{array}\right)+\left(\begin{array}{c} \Delta r_{i}\cos\alpha_{i}\sin\theta_{i}-\Delta x_{i}\cos\theta_{i}\\ \Delta r_{i}\cos\alpha_{i}\cos\theta_{i}+\Delta x_{i}\sin\theta_{i}\\ \Delta r_{i}\sin\alpha_{i}+\Delta z_{i} \end{array}\right)\right]$$

Combining Equations (1) and (3), the 3-D points swept by the *i*-th laser are, in fact, parameterized over a rotating beam:

(4) $$g_{i}(r,\theta )=R_{\theta }(r\cdot a_{i}+\tau_{i})$$

where *a_i_* and *τ_i_* are the direction vector and the origin of the beam, respectively. One can verify that the metric parameter *m_i_* is completely dominated by ‖*a_i_*‖ since ‖(cos*a_i_* sin*θ_i_*, cos*a_i_* cos*θ_i_*, sin*a_i_*)‖ = 1. Thus, all six laser-specific parameters are retained by *a_i_* and *τ_i_*.

We use the derived linear form (*a_i_*, *τ_i_*) instead of the canonical nonlinear parameters to interpret raw measures in Cartesian coordinates. From the experiment results we found that by using linear representation, the optimization process converges faster, and is able to attain a solution with lower error. Furthermore, the evaluation of 3-D coordinates utilizing the linear model is much more computational efficient.

### 3.2. Adjustment of Intrinsic Parameters

A typical calibration of the geometric sensory parameters is based on the observations of particular objects with known geometry. These objects are known as the calibration targets. Since it is fairly common to find planar objects, such as walls or floors, in both outdoor and indoor environments, plane geometry is ideal for the recalibration of Velodyne LiDAR system in this work.

A 3-D plane that does not pass through the origin can be uniquely defined by a 3-vector *n* = (*n_x_*, *n_y_*, *n_z_*) which denotes the point on the plane closest to the origin, as illustrated in Figure 3(b). In the rest of this paper we refer to a plane by its representative vector and *vice versa*. For each point *g*, the signed orthogonal distance to a plane *n* is defined by:

(5) $$\delta (g,n)=\langle g,\frac{n}{\left\|n\right\|}\rangle -\left\|n\right\|$$

where < > and ‖ ‖ are the dot and the 2-norm operators, respectively. It holds that *δ*(*g*, *n*) = 0 if and only if the point is contained in the plane. Suppose the parameters are denote by *β* = {*a*_1_, *τ*_1_, … *a*_64_, *τ*_64_, *n*_1_, *n*_2_, …}, then, for the *i*-th rangefinder, a sub-objective function is defined as:

(6) $$\phi_{i}(\beta )=\frac{1}{2}\sum_{k=1}^{N_{i}}\delta {\left(g_{i}^{\beta }(r_{i,k},\theta_{i,k}),n_{\rho (i,k)}^{\beta }\right)}^{2}$$

where ρ(*i*, *k*) is the index of the calibration plane on which a point $g_{i}^{\beta }(r_{i,k},\theta_{i,k})$ is supposed to lie, and the plane's representative vector is denoted by $n_{\rho (i,k)}^{\beta }$ According to Equation (6), an objective function Φ(*β*) = Σ_1≤_*_i_*_≤64_ *ϕ_i_*(*β*) is designed to minimize the systematic error caused by the intrinsic parameters in terms of plane deviation. The non-linear problem of minimizing Φ is instantiated by taking into account the on-site calibration data (*r_i,k_*, *θ_i,k_*) and $n_{\rho (i,k)}^{\beta }$, which are acquired from the scene. Similar models can also be found in related work [4–6].

### 3.3. Uniqueness of Optimal Solution

One may notice that the term $n_{\rho (i,k)}^{\beta }$ in Equation (6) is also parametrized by *β*. That is because the calibration planes are initially estimated from the point cloud measured by the LiDAR system; hence they should be adjusted as $g_{i}^{\beta }$ updates. Since the dependency cannot be removed unless the planes are estimated from other data sources, it has been suggested to manipulate the calibration planes as adjustable parameters [5,6]. However, deploying the plane-adjustable minimization model also introduces trivial solutions to Equation (6). We observed that if the stopping criterion is not carefully set, the optimization will eventually reach a global minimum where Φ(*β*) ≅ 0 no matter how the parameters are initialized. At a global minimum, all calibration planes are orthogonal to the rotation axis and all points are collapsed to the planes, which is obviously an invalid configuration.

To avoid approaching a trivial solution while maintaining the dependency, we allow the planes to be adjusted within a controllable region. To this end, the parameterization of *j*-th calibration plane $n_{j}^{\beta }$ is defined as:

(7) $$n_{j}^{\beta }=n_{j}^{0}+\Delta n_{j}^{\beta }$$

subject to the constraint $\left\|\Delta n_{j}^{\beta }\right\|\leq \Delta n_{j}^{\max}$, where $0\leq \Delta n_{j}^{\max}<\infty$ is the restricted magnitude of the update. The value of $\Delta n_{j}^{\max}$ can be determined by the confidence of the estimation of $n_{j}^{0}$.

Another issue that needs to be dealt with is the fact that the adjustment can be ill-posed under certain conditions. For the *i*-th rangefinder, if all of its calibration planes are parallel to the rotation axis (z-axis), then ∂*ϕ_i_*(*β*)/∂*a_it_* and ∂*ϕ_i_*(*β*)/∂*τ_it_* will all be zero. As a result, the optimization turns into an ill-posed problem, with infinite many solutions. Similarly, when the calibration planes are all orthogonal to the rotation axis, the partial derivatives of *ϕ_i_* with respect to parameters {*a_ix_*, *a_iz_*, *τ_ix_*, *τ_iz_*} will be zeros. To summarize the issues in this section for ensuring the uniqueness of the optimal solution, the following list provides the problems that should be avoided and how to avoid them in calibration:

- *Trivial solutions in optimization*—Avoided by only allow the planes to be adjusted within a controllable region subject to the constraint $\left\|\Delta n_{j}^{\beta }\right\|\leq \Delta n_{j}^{\max}$.
- *Calibration planes parallel/orthogonal to the rotation axis (z-axis)*—Avoided by tilting the scanner by a predetermined angle.

## 4. Automatic Establishment of Calibration Data

In this section we describe the establishment and processing of calibration data for the adjustment of parameters. In our work, the calibration data are essentially point-plane correspondences that are extracted from the on-site scans. In order to precisely detect planes, the preprocessing on range data as described in Sections 4.1 and 4.2 are carried out. In Section 4.3 we introduce an automatic plane detection algorithm, which is applied to the point cloud constructed from the preprocessed range data. The methods presented in this section allow the calibration data to be acquired in a fast, yet precise manner.

### 4.1. Spatial-Temporal Sensory Data Fusion

In the raw measurements, we discovered noticeable temporal instability that causes scattering of range data, as shown in Figure 1. The instability is identified in both range and angle measurements. In a static scene the deviation of measured ranges is about 2.5 cm over time, which is higher than manufacturer's specification. In addition, a slight quantization error of rotation angle, which is estimated in [5] to be around 0.02°, also affects the accuracy of collected data. In addition, the angular interval of measurement is not guaranteed to be a constant. For instance, when the system operates at 600 rpm the difference in angle between two consecutive measurement can be 0.09°, 0.18°, 0.27°, or any multiple of 0.09°. These hardware issues must be addressed before precise calibration data can be established.

A spatial-temporal data fusion technique is deployed to integrate raw measurements of multiple spins into more reliable range data. We intend to use continuous range data instead of data acquired from a single spin. The resulting data fusion algorithm is based on the idea of estimating a point using a convex combination of adjacent points. The estimated value at point *p* is given by:

(8) $$\hat{r}(p)=\sum_{\forall q\in \text{adj}(p)}\omega_{\text{pq}}\cdot r(q)$$

where *ω_p,q_* is a non-negative weight and $\sum_{\forall q\in \text{adj}(p)}\omega_{\text{pq}}=1$. In our case, *r* is a 2-D spatial-temporal structure containing raw distance measured by a laser rangefinder, and the position, *p* = (*θ*, *t*), represents the rotation angle and the time of measurement. A similar idea is found in the up-sampling algorithm introduced in [9].

Since each laser rangefinder operates at a high speed rate of around 20,000 fires per second, the computation of Equation (8) could be the performance bottleneck. To facilitate the computation, we group all data returned in the same spin and assume that they are measured simultaneously. Although the assumption may not be valid in mobile sensing applications, it should be reasonably acceptable if the LiDAR system is stationary.

**Figure 4.**
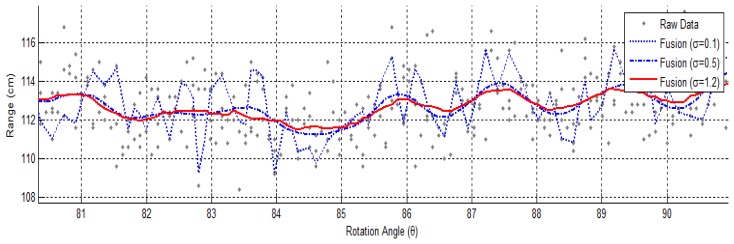
Kernel-based Range Data Fusion with Various σ.

In this work the weights are determined by the Gaussian distance *ωpq* = *e*^−‖^*^p^*^−^*^q^*^‖2^/2*^σ^*^2^. The estimation can be efficiently computed using separable 2-D convolution. To exclude the effect of missing data a normalization term is added to Equation (8):

(9) $$\hat{r}(\theta ,t)=\frac{\left(R*K_{\text{Gauss}}^{\sigma })(\theta ,t\right)}{\left(M*K_{\text{Gauss}}^{\sigma })(\theta ,t\right)}$$

where * is the convolution operator, $K_{\text{Gauss}}^{\sigma }$ is the Gaussian kernel of standard deviation σ, *R* is the raw data, and *M* is the binary function defined as *m*(*θ*, *t*) = 1 if *r*(*θ*, *t*) is valid and *m*(*θ*, *t*) = 0 otherwise. The size of $K_{\text{Gauss}}^{\sigma }$ and the parameter σ are adjustable to control the number and significance of data used for the computation of r̂ (*θ*, *t*). Figure 4 shows a result of fused range data obtained from 6 spins.

Since $K_{\text{Gauss}}^{\sigma }$ is a linearly separable kernel, the data fusion algorithm defined by Equation (9) can be implemented in real-time using fast 1-D convolution. With the optimized circular data structure and the pre-computation of convolution kernels, our CPU-based parallel implementation is able to fuse more than 3 million points per second on a 3.0 GHz quad-core processor. The computation rate is comparable to the firing speed of the LiDAR system. A higher frame rate is possible for a GPU implementation, as illustrated in [9].

### 4.2. Range Segmentation

We apply a derivative-based approach on fused range data to detect points that are continuous and thus likely to be in the calibration planes. The approach works as follows. An *n*-th order Laplacian operator is applied on the fused data. Significant discontinuities are identified by checking the derivative at each angle. By taking the discontinuous points as endpoints and connecting them in a piece-wise manner, we obtain the segmented range profile.

The result is further examined to exclude short segments, which are less likely to contain useful data for the calibration. As a result of removing these outliers, which are the majority of the acquired range data, the detection time of plane is dramatically reduced. Since the segments are detected in the angle-range domain, they may be curved in 3-D space. Despite the curvature, these points are still useful as long as they are planar points.

### 4.3. RANSAC-Based Plane Detection

Finding planes in a point cloud has been a fundamental problem in the field of 3-D modeling. One of the classical solutions is Hough Transform, which searches objects of a particular geometry by means of the accumulator in the parameter domain [10]. The exhaustive construction of the accumulator is, however, very time consuming. A scan like the one we used for the calibration usually contains hundreds of thousands of points. In such case, the time required by Hough Transform could be intolerable for the on-line calibration process. Furthermore, the layered misalignment of points also makes it more difficult to find concentrated distribution of plane scores in the accumulator. Thus, we need a heuristic and robust algorithm to locate the calibration targets efficiently.

A RANdom SAmpling Consensus (RANSAC) technique that iteratively searches calibration planes in a stochastic manner is developed to locate the calibration targets efficiently. In each round, a point and some of its neighbors are randomly selected. Based on the selected points a best-fitting plane is calculated to minimize residuals in a least-square sense. This plane is then applied to measure its fitness in the global scope with respect to whole point cloud.

**Figure 5.**
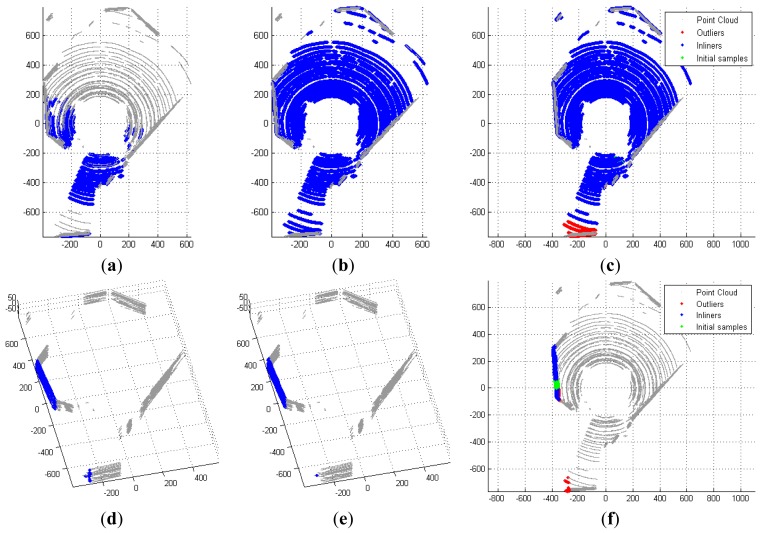
(**a**) initial estimate; (**b**) refined estimate using ICP-based algorithm; and (**c**) final result of the detection of floor surface; (**d**–**f**) the same process applied to find another surface.

For an acceptable estimate, which contains a significant number of points within a tolerable deviation ∈, we refine the plane using the Iterative Closest Point (ICP) technique as follows. Firstly, the points that are likely to be in the plane within tolerable deviation are selected. A least-square plane is then calculated subject to these points to replace the initial estimate. The refinement repeats until termination criteria are met that either the update of the plane is significantly small or the number of iterations reaches its limit. Points in the estimated plane are removed from the point cloud, and the new estimate is added to the list of detected planes. Figure 5 shows two examples of the detection process.

Since the selection of point is done in the global scope, some points contribute to the estimate may be outliers that are far from the majority. Figure 5(d) shows the inclusion of such outliers. To address this issue, we apply Principle Component Analysis (PCA) on the Cartesian coordinates and study the distribution of the first two principle components of the selected points. The points that are significantly distant from the majority are excluded. In Figure 5(d–f) one can see a portion of the points on the smaller wall are taken into account for the initial estimate and excluded in the final result.

**Figure 6.**
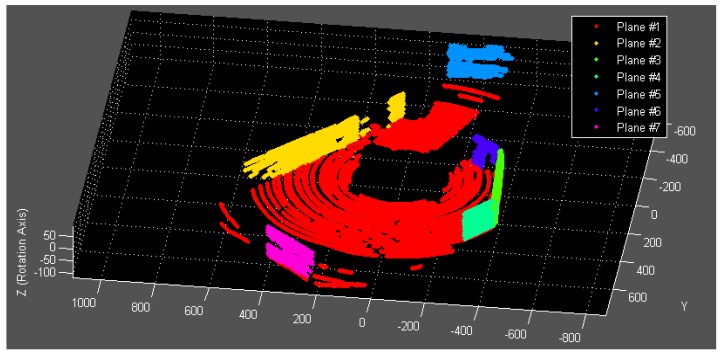
Established calibration data containing seven planar surfaces.

This stochastic process iterates until no more planes can be found or the number of iteration reaches its maximum. The pseudo-code of the algorithm is listed in Algorithm 1 and Algorithm 2. The established point-plane correspondences (see Figure 6 for an example) are now qualified to be used as calibration data for the optimization process described in Section 3.

**Algorithm 1.** Algorithm of RANSAC-based plane detection.
**FindPlanesRansac**
***Input:***
*Point cloud G*, *sampling ratio σ*, *positive ratio of acceptance ρ*, *error tolerance ∈*, *number of iterations k****Output:***
*Set of detected planes N*
1*N* ← {∅}2For *i* = 1 to *k*3Draw a sample *g_i_* ∈ *G* and *σ* samples *S_i_* ∈ *G* in the vicinity of *g_i_*4*n_i_* ← *BestFitPlane*(*S_i_*)5*P_i_* ← {*p* ∈ *G: δ*(*p*, *n_i_*) < *ε*}6If |*P_i_*|/|*G*| > *ρ*7*n_i_* ← *RefinePlaneICP*(*P_i_*, *n_i_*, ∈, *P_i_* ←{*p* ∈ *G: δ*(*p*, *n_i_*) < *ε*}8*N* ← *N* ∪ {*n_i_*}, *G* ← *G* − *P_i_*9End If10End For


**Algorithm 2.** Algorithm of ICP-based plane refinement.
**RefinePlaneICP**
***Input:***
*Point cloud G*, *initial plane n*_0_, *error tolerance ∈*, *number of iterations k****Output:***
*Refined plane n_k_*
1*n*_0_ ← *BestFitPlane*(*P_i_*)2For *i* = 1 to *k*3 *P_i_* ← {*p* ∈ *G : δ*(*p*, *n_i_*_−1_) < *ε*}, *n_i_* ← *BestFitPlane*(*P_i_*)4 If ‖*n_i_* − *n_i_*_−1_‖ is small5  *n_k_* ←*n_i_*, *i* ← *k* // early stop6 End If7End For

## 5. Experimental Results

### 5.1. On-Site Data Acquisition

**Figure 7.**
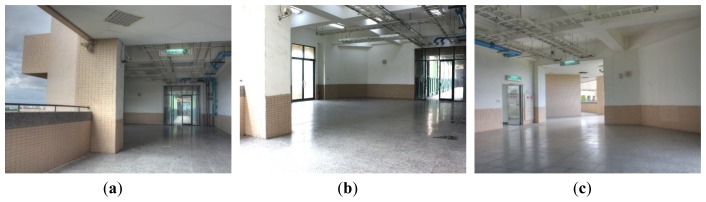
The corridor selected to evaluate the proposed method.

The range data are collected in an open space to evaluate the proposed method. The selected site, as shown in Figure 7, is an outdoor corridor located on the fourth level of a campus building. There are eight angled walls and one tiled floor in the scene. We place the LiDAR system in three different positions for data acquisition. According to the issues stated in Section 3.3, the scanner is tilted by 30° in the second and third positions to ensure the uniqueness of optimal parameters. Three calibration datasets are automatically established using the algorithms introduced in Section 4. The parameters used to detect planes are set as *σ* = 1.0, *ρ* = 0.3, *∈*, and *k* = 100. Summary of these datasets are listed in Table 1. The factory parameters are converted to the linear form and optimized using the Levenberg-Marquardt method with numerical approximation of first and second order derivatives. The adjustment of calibration planes is constrained within a radius of 2.5 cm centering on its initial value.

### 5.2. Evaluation of LiDAR Recalibration

**Figure 8.**
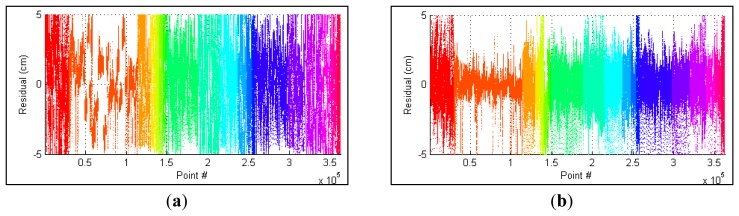
Point residuals colour-coded to show calibration planes (**a**) factory parameters; (**b**) recalibrated parameters.

**Figure 9.**
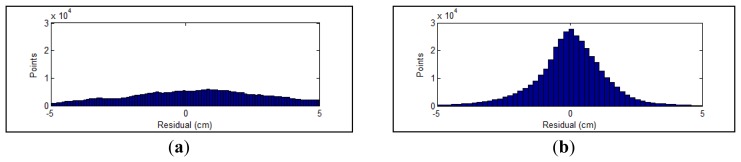
Distribution of point residuals (**a**) factory parameters; (**b**) recalibrated parameters.

**Figure 10.**
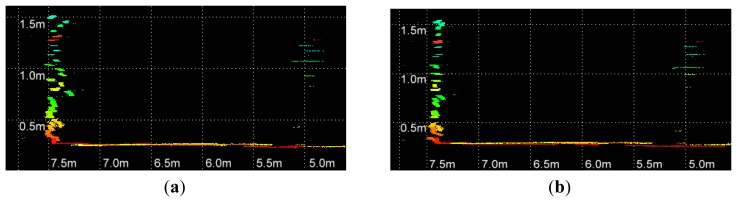
Scan of a wall (**a**) before recalibration; (**b**) after recalibration.

The residual between each point and the corresponding calibration plane is measured to evaluate the performance of parameters. In Figure 8, the errors of parameters recalibrated using all collected datasets are compared with the factory parameters. Before the optimization, the RMS and standard deviation of calculated residuals are 2.39 cm and 2.37 cm, respectively. The recalibrated parameters achieve a RMS of 1.39 cm with a standard deviation of 1.39. The RMS error is decreased by 42% after the adjustment. The distributions of point residuals are depicted in Figure 9. A visually observable result of the improvement is given in Figure 10, which shows views orthogonal to a scanned wall. As can be seen from Figure 10, the layered misalignment among measurements returned by different rangefinders is reduced significantly. Please note that for each layer the scattering caused by the measurement errors of around ±25 mm still presents.

Cross validation is also conducted by selecting some subsets of three calibration datasets to perform the recalibration, and evaluating the result using datasets that are not used during the optimization. For an unused dataset, the planes are re-estimated from the point cloud using the optimized intrinsic parameters. The results are given in Table 2. The shaded cells indicate that the corresponding dataset is not taken into account to adjust the parameters. The evaluation on these unused datasets usually contributes to a slightly higher error, which is still below the baseline. It shows that the result of on-site recalibration outperforms manufacturer-calibrated parameters, and the improvement ranges from 14% to 50%.

### 5.3. Comparison of Linear and Non-Linear Parameters

**Figure 11.**
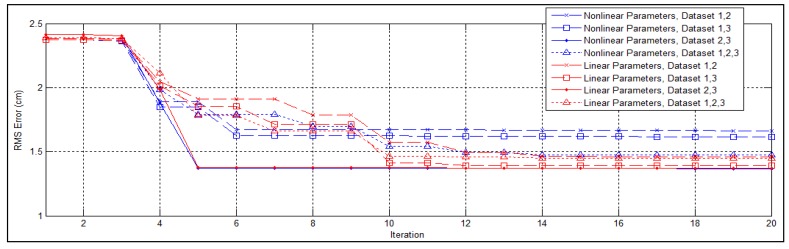
Convergence of different parameter representations.

To examine the effects of representing the intrinsic parameters in the linear form derived in Section 3.1, we conduct the same experiment using the non-linear representation defined by the manufacturer. The traces of RMS error through first 20 iterations of the optimization process are shown in Figure 11. In three out of four cases, the linear parameters converge earlier than the non-linear ones. In two cases the optimization failed to converge within 100 iterations when the non-linear parameters are adopted. The experimental results also indicate that the optimal solutions obtained using the linear representation achieves lower overall error, with the maximal improvement of 35%.

Similar to [5], we also provide the correlation matrix of the estimated parameters to examine the accuracy of the estimation. The correlation data are obtained by calculating the inverse of the Hessian matrix, which is numerically approximated by means of the Jacobian matrix at the optimal solution. Tables 3 and 4 tabulate the averaged correlation coefficients of the estimated parameters over all four tests. The bold numbers indicate significant correlations. The higher-than-median correlations are shaded, and the highly correlated observations are marked with thicker borders. The asymptotic standard errors are also listed in the tables to study the certainty of estimation.

As we expected, strong correlations are found between the linear parameters (*a_x_*, *τ_x_*), (*a_y_*, *τ_y_*), and (*a_z_*, *τ_z_*), which are respectively related to the same directions. However, the overall correlation is lower than the canonical parameters. In other words, the behavior of non-linear parameters is less decoupled than that of the linear parameters. This may explain the observation that the minimization solver approaches an optimal estimate quicker when the linear form is adopted.

### 5.4. Effects of Data Fusion

The fusion of range measurements reduces the amount of computation. To verify how the reduction of data affects the result of calibration, we conduct the same test, but this time with all calibration datasets established from 894,000 raw measures. The results are compared with the use of fused range data consisting of 377,000 points to study the difference. The RMS error of raw range data is higher than the results listed in Table 2. However, it is not appropriate to use point-plane residuals for evaluation since the range data are not identical. The comparison instead refers to the misalignment of adjusted planes. The evaluation estimates the plane-based optimal rigid transformation of the LiDAR system between the sites where Dataset 2 and Dataset 3 are collected. The misalignment of planes is then measured in terms of angular and distant error, as listed in Table 5.

The evaluated errors with and without the fusion of range data is barely distinguishable compared to the errors of factory parameters. The largest differences are 0.08° in angle and 0.164 cm in distance. However, there exists noticeable difference in the running time. The optimization on the fused range data finished in 517 s. In contrast, without the fusion the optimization took 3,132 s to converge. The reduction of data greatly boosts the process of recalibration while achieving a similar result. Yet another observation is that the misalignment of planes is reduced even though it is not explicitly modeled by the objective function.

## 6. Conclusions and Future Work

We present in this work an efficient multi-stage strategy to attain automatic on-site recalibration of the Velodyne HDL-64E S2 system. The proposed method is applicable to both range-angle sensory space as well as Euclidean space. In the first stage, the range data are merged temporally and spatially using a real-time Gaussian-based algorithm. The amount of range data is then reduced by enforcing the continuity constraint. Afterward, we carry out a robust RANSAC-based plane detection algorithm to locate planes in 3-D space. The estimated planes are refined in an ICP manner before the final calibration dataset is established. The intrinsic parameters optimized using the on-site range data achieve an average improvement of 40% over the factory parameters. In the experiment, the plane residuals with a RMS error lower than 1.3 cm is attainable, which is an improvement from previous work. The implementation of the on-site calibration strategy also allows the LiDAR to be automatically calibrated before each acquisition sequence, such that system can use calibration parameters that are more adapted to each individual site for obtaining more accurate results.

A linear form of the parameters is also derived from the range data conversion formula specified by the manufacturer. The parameters represented in the linear form are verified to be less correlated and achieve lower RMS error quicker than the canonical model. The optimization model defined in this paper can be further extended to a LiDAR system that follows the rotating multi-beam model described by the linear parameters. The process is designed to be finished on-site so that one can see and use the improved interpretation of the range data as quickly as possible. For a calibration dataset containing twenty planes with one million points, the whole process (data collection, preprocessing, plane detection, and optimization) can be finished within 10 min on a moderate laptop. Similar to many computer vision applications, we suggest that the calibration process be performed at the beginning of a data acquisition sequence for each different site. For example, calibration is performed once at the beginning for the corridor sequence and the acquired parameters are used throughout the data acquisition for that site. Since the proposed calibration procedure is quick and simple to perform, it is easy to include a calibration before each different range data sequence is taken.

In future work, we will incorporate image sensors into the system to study simultaneous calibration of intrinsic and extrinsic parameters (e.g., [11]). The linear representation of laser parameters also allows further reduction of unknowns in the optimization model for integrating image sensors with the LiDAR system. Finally, once the image sensors have been integrated with the LiDAR system, we wish to investigate the possibility of analysing the laser trajectories from IR images captured by the image sensors, such that we may validate the performance of recalibrated parameters and improve upon our current results.

## Figures and Tables

**Table 1.** Established calibration datasets.

	**Raw Points**	**Fused Points**	**Continuous Pts.**	**Final Points.**	**Reduction**	**Planes**
**Dataset 1**	620,996	219,145	183,501	149,465	76%	8
**Dataset 2**	135,228	175,257	139,687	122,884	10%	6
**Dataset 3**	510,775	174,263	137,715	104,958	79%	6

**Table 2.** RMS Errors and Improvement After Recalibration.

	**Factory Parameters**	**Parameters Optimized using On-site Range Data**			
		**Dataset 1, 2**	**Dataset 1, 3**	**Dataset 2, 3**	**Dataset 1, 2, 3**
**Dataset 1**	2.355 cm	1.323 cm (44%)	1.209 cm (49%)	2.014 cm (14%)	1.285 cm (45%)
**Dataset 2**	2.426 cm	1.532 cm (36%)	1.776 cm (27%)	1.363 cm (44%)	1.491 cm (39%)
**Dataset 3**	2.397 cm	1.733 cm (28%)	1.457 cm (39%)	1.307 cm (45%)	1.412 cm (41%)
**Overall**	2.389 cm	1.493 cm (38%)	1.482 cm (38%)	1.663 cm (30%)	1.386 cm (42%)

**Table 3.** Average correlation matrix and estimated error of linear parameters.

	*a_x_*	*a_y_*	*a_z_*	*τ_x_*	*τ_y_*	*τ_z_*	**Standard Error**
*a_x_*	1.0000	0.0735	0.0598	−**0.9196**	−0.0721	−0.0545	0.0004 cm
*a_v_*	-	1.0000	**0.2100**	−0.0687	−**0.9575**	−**0.2599**	0.0004 cm
*a_z_*	-	-	1.0000	−0.0349	−**0.2033**	−**0.9208**	0.0003 cm
*τ_x_*	-	-	-	1.0000	0.0743	0.0368	0.1170 cm
*τ_v_*	-	-	-	-	1.0000	**0.2702**	0.1621 cm
*τ_z_*	-	-	-	-	-	1.0000	0.0769 cm

**Table 4.** Average correlation matrix and estimated error of non-linear parameters.

	*θ*	*α*	*m*	Δ*x*	Δ*y*	Δ*z*	**Standard Error**
*θ*	1.0000	−0.0679	0.0766	−**0.9542**	−0.0784	0.0641	0.0219°
*α*	-	1.0000	−**0.4290**	0.0509	**0.3168**	−**0.9570**	0.0231°
*m*	-	-	1.0000	−0.0780	−**0.9459**	**0.5169**	0.1765 cm/cm
Δ*x*	-	-	-	1.0000	0.0747	−0.0508	0.1535 cm
Δ*r*	-	-	-	-	1.0000	−**0.3973**	0.1533 cm
Δ*z*	-	-	-	-	-	1.0000	0.0004 cm

**Table 5.** Angular and distance plane misalignment with and without data fusion.

**Plane**	**Factory Calibration**		**Recalibrated with Fusion**		**Recalibrated without Fusion**	
**Index**	**Angular Err.**	**Distant Err.**	**Angular Err.**	**Distant Err.**	**Angular Err.**	**Distant Err.**
**1**	0.2062°	0.6559 cm	0.1794°	0.2743 cm	0.2205°	0.1129 cm
**2**	0.2249°	1.6283 cm	0.1042°	1.4106 cm	0.1520°	1.4961 cm
**3**	0.1659°	0.5163 cm	0.1803°	0.2835 cm	0.0988°	0.2909 cm
**4**	0.1257°	0.7623 cm	0.1266°	0.7604 cm	0.1928°	0.8136 cm
**5**	0.1334°	0.3600 cm	0.1766°	0.2037 cm	0.1449°	0.1942 cm
**6**	0.3623°	0.1985 cm	0.3169°	0.0644 cm	0.2800°	0.0646 cm
**Overall**	0.2031°	0.6869 cm	0.1807°	0.4995 cm	0.1815°	0.4954 cm

## References

[1] Sebastian Thrun: Google's Driverless Car. http://www.ted.com/talks/sebastian_thrun_google_s_driverless_car.html.

[2] Velodyne's Lidar Features in Seven DARPA Robot Vehicles. http://www.traffictechnologytoday.com/news.php?NewsID=1780.

[3] Muhammad N., Lacroix S. Calibration of a Rotating Multi-Beam Lidar.

[4] Atanacio-Jiménez G., González-Barbosa J.-J., Hurtado-Ramos J.B., Francisco J., Jiménez-Hernández H., García-Ramirez T., González-Barbosa R. (2011). Velodyne HDL-64E calibration using pattern planes. Int. J. Adv. Robotic. Syst..

[5] Glennie C., Lichti D.D. (2010). Static calibration and analysis of the Velodyne HDL-64E S2 for high accuracy mobile scanning. Remote Sens..

[6] Glennie C., Lichti D.D. (2011). Temporal stability of the Velodyne HDL-64E S2 scanner for high accuracy scanning applications. Remote Sens..

[7] Bae K.H., Lichti D. On-Site Self-Calibration Using Planar Features for Terrestrial Laser Scanners.

[8] (2008). HDL-64E S2 User's Manual.

[9] Jennifer D., Jongmin B., Christian P., Sebastian T. Upsampling Range Data in Dynamic Environments.

[10] Dorit B., Jan E., Kai L., Andreas N. (2011). The 3D hough transform for plane detection in point clouds—A review and a new accumulator design. J. 3D Res..

[11] Mirzaei F.M., Kottas D.G., Roumeliotis S.I. (2012). 3D Lidar-camera intrinsic and extrinsic calibration: Identifiability and analytical least-squares-based initialization. Int. J. Robot. Res..

